# Sampling methods affect Nematode-Trapping Fungi biodiversity patterns across an elevational gradient

**DOI:** 10.1186/s12866-020-1696-z

**Published:** 2020-01-16

**Authors:** Wei Deng, Jia-Liang Wang, Matthew B. Scott, Yi-Hao Fang, Shuo-Ran Liu, Xiao-Yan Yang, Wen Xiao

**Affiliations:** 1 Institute of Eastern-Himalaya Biodiversity Research, Dali University, Dali, Yunnan 671003 China; 2 Collaborative Innovation Center for Biodiversity and Conservation in the Three Parallel Rivers Region of China, Dali, Yunnan 671003 China; 3 The Provincial Innovation Team of Biodiversity Conservation and Utility of the Three Parallel Rivers Region, Dali University, Dali, Yunnan 671003 China; 4 The Key Laboratory of Yunnan Education Department on Er’hai Catchment Conservation and Sustainable Development, Dali, Yunnan 671003 China; 5 Fu Yang People’s Hospital Infection Management Section, Fuyang, Anhui 236000 China; 6 Scion (New Zealand Forest Research Institute), Christchurch, 8011 New Zealand

**Keywords:** Elevation richness gradient, Microbial distribution pattern, Sampling effect, Observation bias, Human disturbance, Biodiversity

## Abstract

**Background:**

Understanding the patterns of species richness across elevational gradients is a key concept for contemporary research in ecology and evolution, and critical to understanding large-scale trends in biodiversity, global change and conservation. However, patterns of elevational species richness between taxonomic groups, regions and latitudes are inconsistent, so that various, sometimes conflicting hypotheses exist. Several scholars have pointed out that research on elevational distribution patterns is often biased by the sampling design employed. To test this hypothesis, we analyzed species richness of Nematode-Trapping Fungi (NTF) across an elevation gradient at two mountainous sites in western Yunnan Province, P.R. China. We tested for potential differences in the results when using different sampling designs.

**Results:**

A total of 3 genera, 17 species, 222 strains of NTF were isolated and identified from Gaoligongshan and Cangshan. Species accumulation curves for both sites and sampling modes had acceptable leveling, demonstrating sufficient sampling effort. At Gaoligongshan, the elevation distribution patterns of NTF were different under two sampling patterns. When reducing the analyzed altitude range in Gaoligongshan, the elevation distribution pattern of the NTF changed. A similar elevation distribution pattern was observed in Cangshan when testing the same altitude range. In general, when treating the same dataset using different sampling designs, the resulting distribution patterns of species richness and occurrence frequencies were clearly different. Moreover, after removal of the samples located within lower-altitude zones affected by anthropogenic interferences, the distribution pattern of NTF in the two sites tended to become uniform.

**Conclusion:**

The sampling design, and in particular the elevation interval between plots, has a significant effect on the assessment of species distribution in mountainous regions. Other factors such as human activities and the multi-dimensionality of biodiversity also contribute to result biases. It is recommended that sampling design is given careful consideration in future studies on the elevational gradients of species richness, using stratified approaches according to the most relevant factors.

## Background

Mountain environments are widely recognized as containing highly diverse and species rich ecosystems [1]. Life zones are condensed over short distances, and patterns of diversity are driven by steep environmental gradients. Patterns of diversity along altitudinal gradients have attracted the attention of scientists for centuries, contributing to ecological niche theory, global life zones, community assembly and insular biogeography [2]. Current research in the subject contributes to our understanding of large-scale trends in biodiversity, global change and conservation.

Despite the extensive research in the field, dating back to Carl Linnaeus, establishing universal theoretical models to explain species richness patterns along elevational gradients has proven challenging and lacks consensus [2–6]. Studies documenting elevational diversity patterns across a wide variety of taxonomic groups, biogeographic regions, and latitudes [4] exhibit four principal patterns of diversity: decreasing, low-elevation plateau (LEP), low-elevation plateau with a mid-peak (LPMP), and mid-peak [7–9]. Other patterns, including bimodal patterns, multi-peak patterns, U-shaped patterns and some irregular patterns, have also been reported [10–12]. However, of these, mid-elevation peak (including LPMP and mid-peak) and decreasing are the most common patterns observed [13, 14].

Elevational patterns of species richness are proposed to be driven by potentially-interacting factors relating to climate (e.g., temperature, rainfall, soil productivity, humidity and cloud cover), space (e.g., species-area relationship, mid-domain effect), evolutionary history (e.g., speciation rates, extinction rates, clade age and phylogenetic niche conservatism, biotic processes (e.g., ecotone effects, competition, mutualisms, habitat heterogeneity and habitat complexity), and more recently, anthropogenic disturbances [4, 15–17]. However, some researchers have suggested some reported elevational distribution patterns are simply a product of a biased sampling pattern [4], citing factors such as sampling pattern, scale and disturbance influence outcomes [2]. Nogués-Bravo et al. [18] showed that truncating the elevational range and extent of the sample gradient affected the observed pattern, and Grytnes and Romdal [19], using rarefaction curves, demonstrated sampling intensity also affects the resulting pattern. Yet, the effects of sampling patterns and range have not been sufficiently identified.

In this study, we measured the diversity and occurrence frequency of Nematode-Trapping Fungi (NTF) on two mountain areas to test whether the pattern would be consistent with the leading hypotheses. We were also interested in testing whether patterns of diversity in NTF could be biased through sampling pattern, intensity, or anthropogenic disturbance, addressing the hypotheses set out by Nogués-Bravo et al. [18] and Grytnes and Romdal [19]. We elected to use NTF because there are limited species numbers in the environment, they are relatively easy to identify, and because we know very little about how NTF diversity varies across elevational gradients. The Three Parallel Rivers Region is a global hotspot for biodiversity characterized by multiple parallel north-south oriented high mountain chains with dramatic altitudinal gradients. We chose two mountain ridges with similar altitudinal ranges as sites to test the effects of elevational range, extent and sampling intensity on elevation diversity pattern of NTF. We hypothesize that sampling strategy affects the observed elevational distribution pattern of NTF, leading to biases on their perceived distribution.

## Results

### NTF species distribution in the study areas

**Fig. 1:**
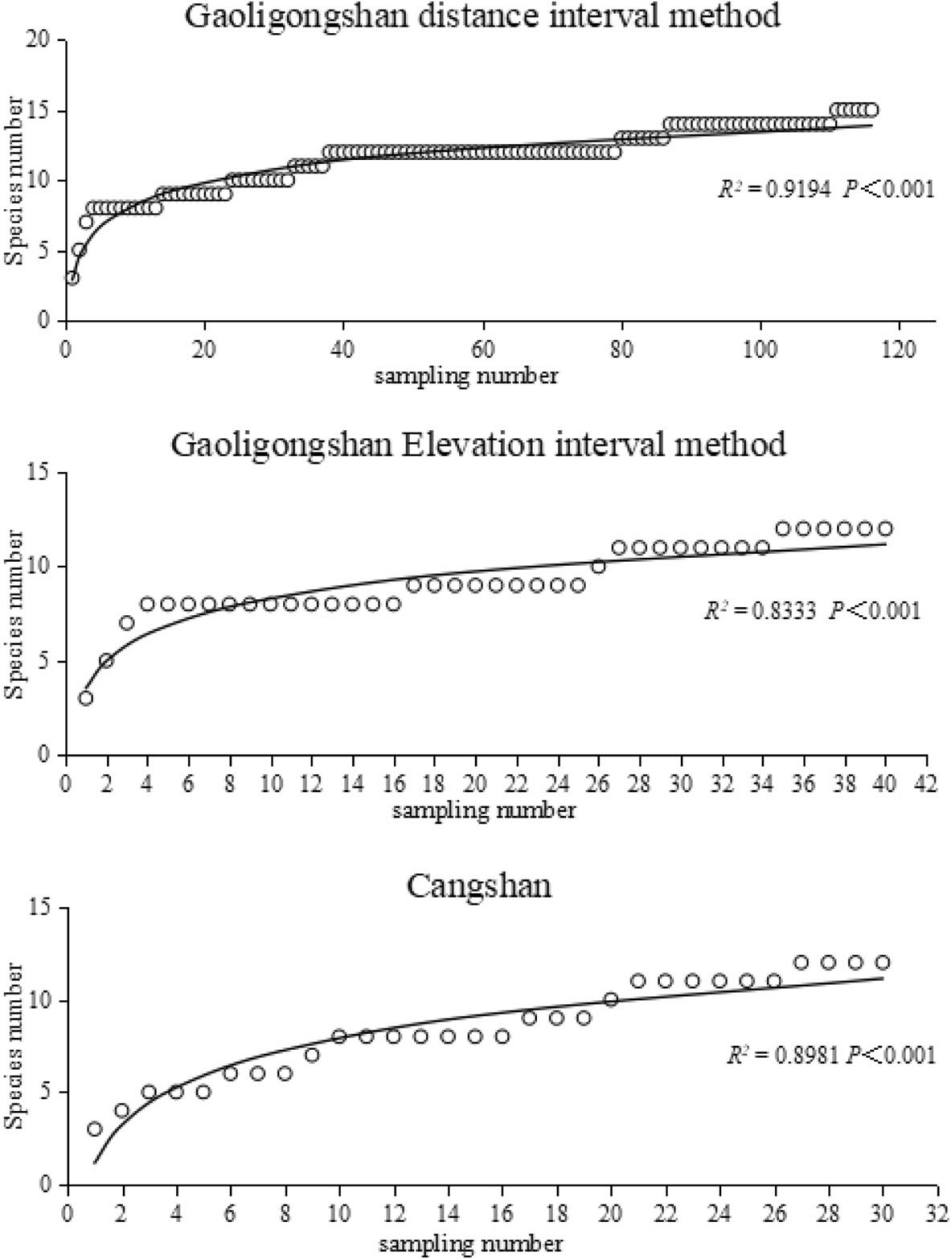
The OF of the NTF in Gaoligongshan showed different distribution patterns in two sampling modes

A total of 3 genera, 17 species, 222 strains of NTF were isolated and identified from the Gaoligongshan and Cangshan sites (Table 1). At Gaoligongshan, *Arthrobotrys thaumasia* was the most widespread and abundant species, occurring in 25% soil samples (Occurrence Frequency, OF = 25%). *Dactylellina drechsleri, Arthrobotrys elegans, Arthrobotrys eudermata, Arthrobotrys javanica* had the narrowest altitudinal range, at Gaoligonshan, found at only one altitude. At Cangshan, *Arthrobotrys musiformis* was the most abundant species (OF = 82%), but *Dactyllina ellipsosporum* had the widest distribution. *Arthrobotrys thaumasia, Arthrobotrys guizhouensis, Arthrobotrys microscaphoides* had the narrowest altitudinal range (Table 1). Species accumulation curves for both sites and sampling modes showed acceptable leveling, demonstrating sufficient sampling effort (Fig. 1).

**Table 1:** Species list and elevational range of NTF obtained from the Gaoligongshan and Cangshan

Species	Gaoligongshan		Cangshan	
	OF(%)	Range(m)	OF(%)	Range(m)
*Arthrobotrys thaumasia*	25.00%	1400–3400	3.33%	2100
*Arthrobotrys oligospora*	22.50%	1400–3000	30.00%	2100–2700
*Arthrobotrys musiformis*	11.67%	1400–2800	81.82%	2200–3100
*Arthrobotrys conoides*	9.17%	1400–2600	13.33%	2200–2700
*Dactylellina ellipsosporum*	7.50%	2000–2800	36.67%	2100–3100
*Arthrobotrys fusiformis*	3.33%	2000–2400	10.00%	3000–3200
*Arthrobotrys robusta*	3.33%	1400–2400	6.67%	2500–2700
*Arthrobotrys microscaphoides*	3.33%	1400–2800	3.33%	3200
*Arthrobotrys guizhouensis*	2.50%	1400–2200	3.33%	2300
*Arthrobotrys rutgeriense*	1.67%	2600–2800	–	–
*Arthrobotrys elegans*	1.67%	1900	–	–
*Arthrobotrys javanica*	1.67%	2600	–	–
*Arthrobotrys eudermata*	0.83%	3100	–	–
*Dactylellina drechsleri*	0.83%	2100	6.67%	2100–2600
*Dactylellina parvicolla*	–	–	6.67%	2900–3000
*Drechslerella aphrobrochum*	–	–	6.67%	2500–2700

### NTF elevational diversity pattern under two sampling patterns at Gaoligongshan

**Fig. 2:**
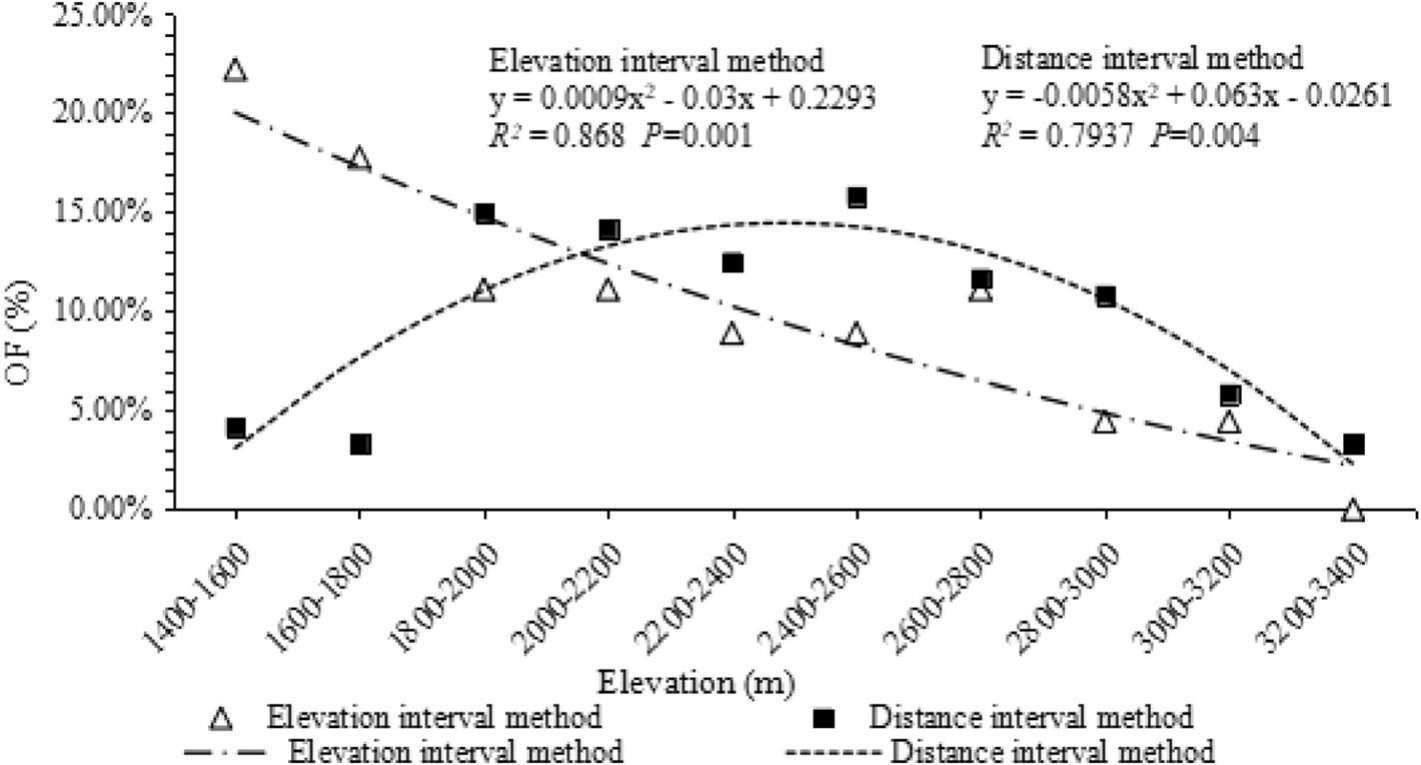
Curves show a decreasing pattern using elevation interval method, and a LPMP pattern using the distance interval method

**Fig. 3:**
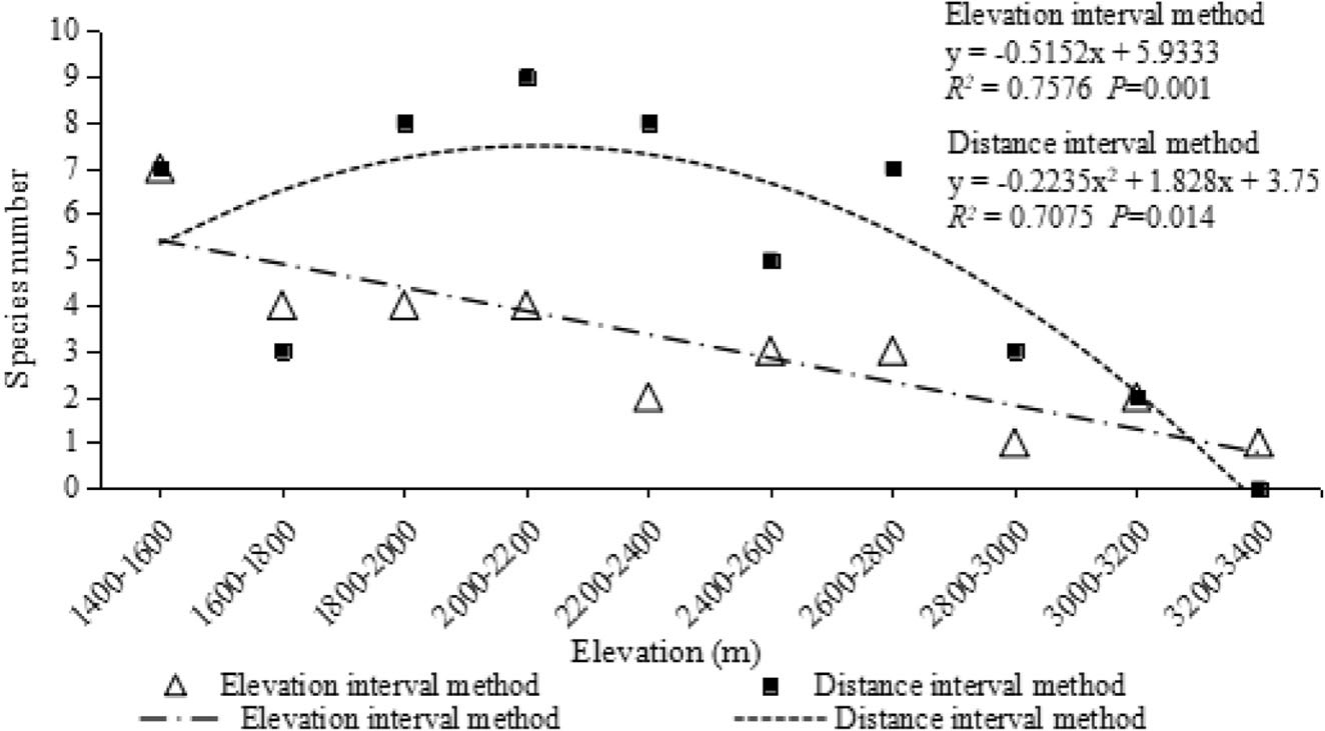
In distance interval method, samples below 2100 m were cut off, and the OF and number of species of NTF showed a decreasing pattern

There were 15 species (120 strains) of NTF isolated and identified from 116 soil samples collected equidistantly along the sampling lines (distance interval method), and 12 species (45 strains) of NTF isolated and identified from 40 soil samples collected along an elevational gradient (elevation interval method). The elevational distribution patterns of NTF were different in two different sampling modes (Fig. 2). In the distance interval sampling method, Occurrence Frequency (OF) showed a mid-peak pattern, whereas the elevation interval sampling method showed a decreasing pattern in along the elevational gradient. For species richness, the distance sampling method showed a LPMP pattern for NTF richness and a decreasing pattern in the elevation sampling method (Fig. 3). The sample sites were unevenly distributed along the sampling lines in the distance sampling methods; the layout of sampling sites had a significant correlation with species distribution (*r* = 0.872, *P* = 0.01).

### The elevational distribution pattern of NTF with smaller altitude range of sampling at Gaoligongshan

**Fig. 4:**
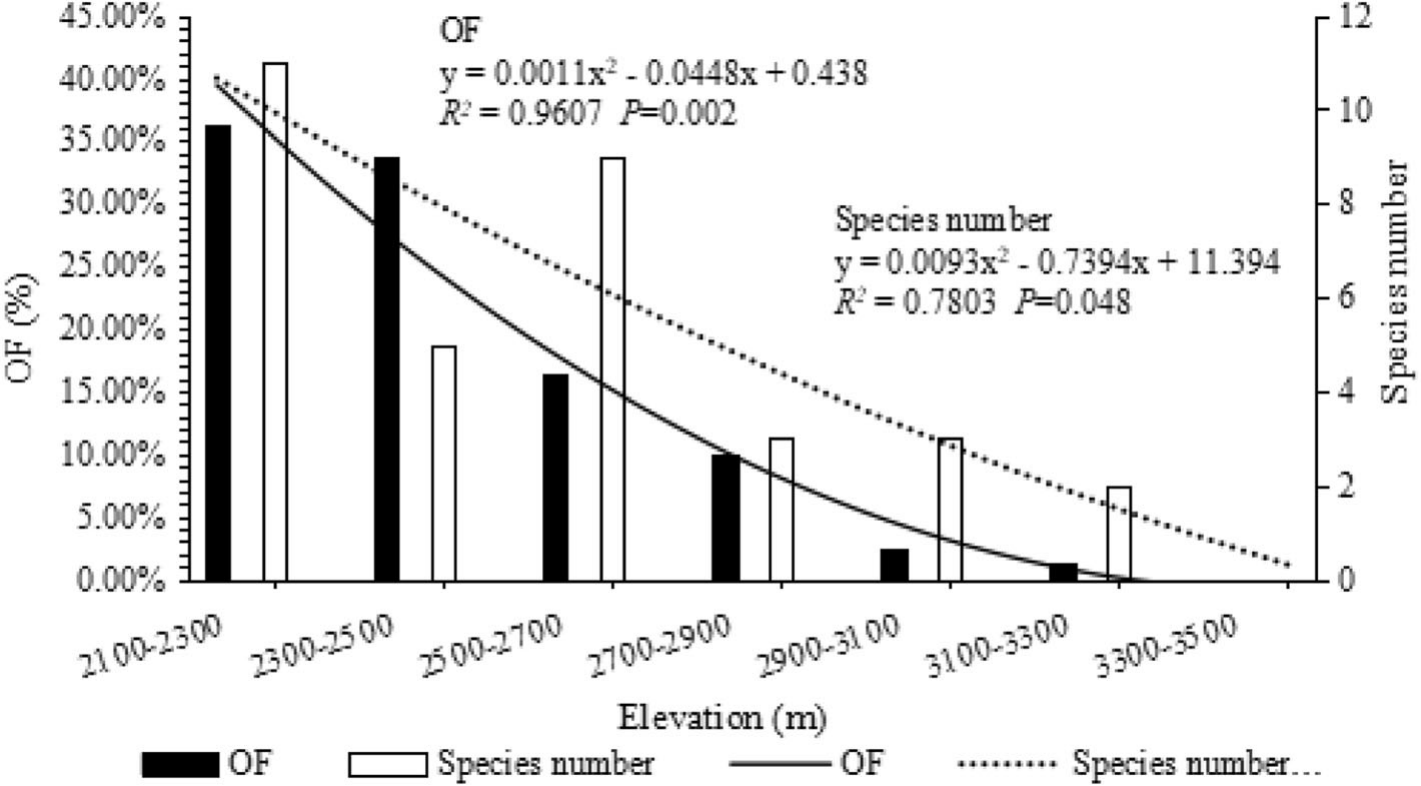
The OF of the NTF in Cangshan shows a decreasing pattern, and the number of species shows a LPMP pattern

Looking at a truncated range (2100–3500 m), the NTF showed some elevational distribution features that the OF and species number of NTF decreased with the increasing elevational gradient, fitting the decreasing pattern (Fig. 4).

### The elevational distribution pattern of NTF at Cangshan

**Fig. 5:**
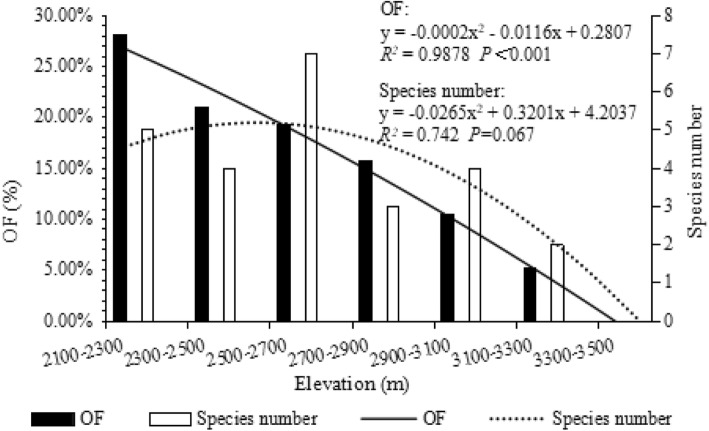
Cangshan (OF: y = −0.0087x + 0.2376, *R*^*2*^ = 0.9756, *P*<0.001; species number: y = 0.0008 × ^3^–0.0476 × ^2^ + 0.5689x + 3.4904, *R*^*2*^ = 0.7546 *P* = 0.140); Gaoligongshan (OF: y = 0.0007 × ^2^–0.0322x + 0.356, *R*^*2*^ = 0.9853, *P* = 0.002; species number: y = 0.0018 × ^3^–0.0845 × ^2^ + 0.8412x + 4.3318, *R*^*2*^ = 0.7314, *P* = 0.195). Pearson correlation of Cangshan and Gaoligongshan (OF:*r* = 0.907, *P* = 0.005; species number: *r* = 0.857, *P* = 0.014)

At Cangshan, there were 12 species (57 strains) of NTF isolated and identified from samples. Occurrence Frequency displayed a decreasing pattern, whereas species number conformed more to a LPMP pattern (Fig. 5).

### The elevational distribution of NTF after removal of the human interference range

**Fig. 6:**
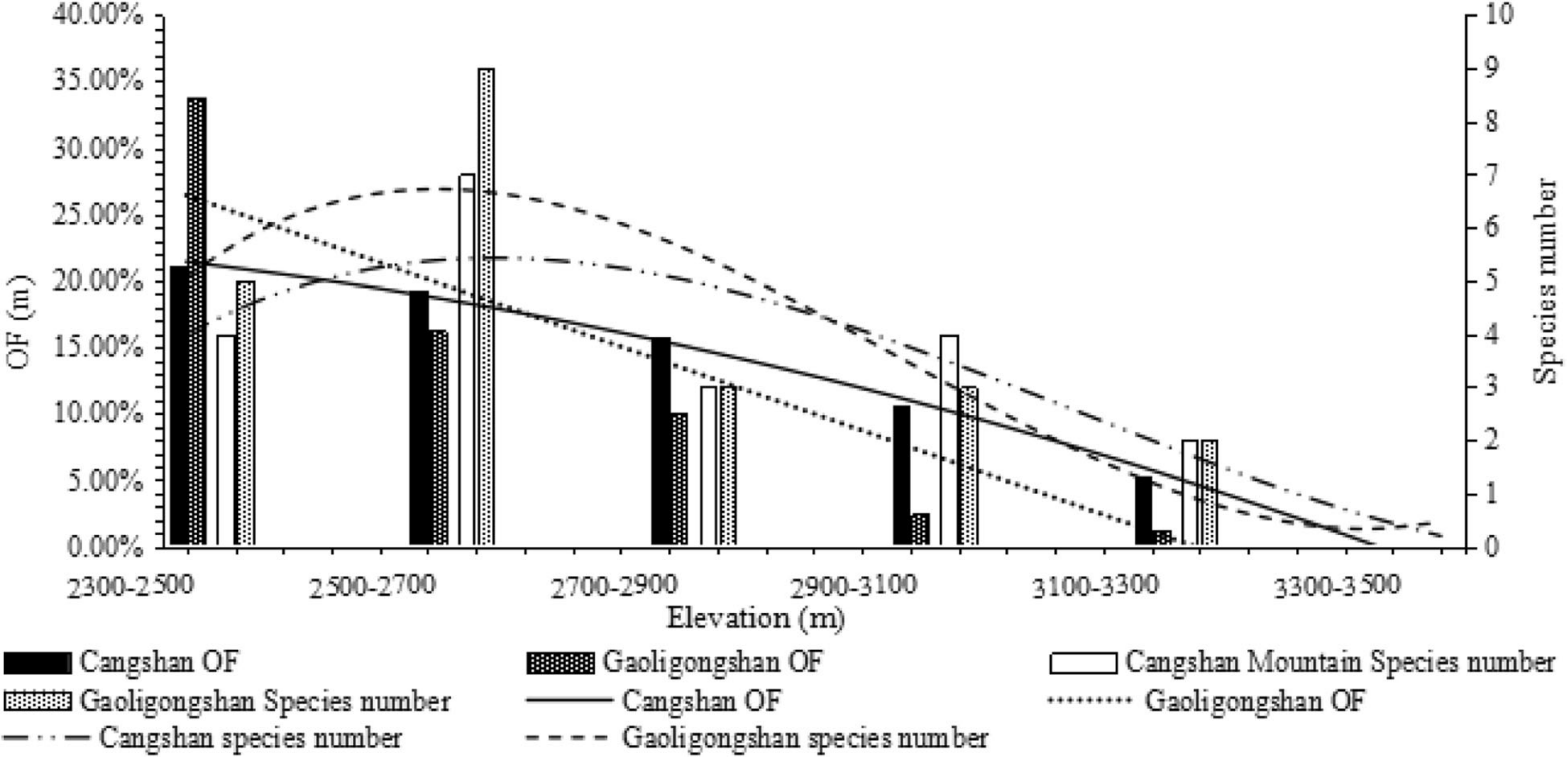
The data are fitted with a binomial fitting model, showing the pattern of the middle peak distribution

After removing data from disturbed lower elevations, OF and species number of NTF had a decreasing pattern at both sites, see Fig. 6. The OF and species number of the two areas were correlated, OF:*r* = 0.907, *P* = 0.005, species number: *r* = 0.857, *P* = 0.014.

## Discussion

### NTF elevational species richness and occurrence frequency pattern

In this study, we compared NTF species richness and occurrence frequency across an elevation gradient on two mountain ranges. Overall, we found species composition similar between the sites, with 11 of the 17 species collected in common. However, we found species abundance and elevational range differed between the sites; previous studies of NTF on Cangshan found community-level structural differences at different altitudes and aspects [20]. In our study, we found diversity of NTF on Cangshan and Gaoligongshan showed mid-elevation peak and decreasing patterns, consistent with many studies on species diversity across an elevation gradient.

While this finding contributes to the growing body of evidence for monotonic (decreasing) or unimodal (mid-elevation peak) diversity patterns found along elevational gradients, it does not provide an explanation for its occurrence. In general, it is quite possible any combination of universal interacting factors, such as climate, space, evolutionary history or biotic process to be driving the observed patterns in NTF diversity across elevation. Patterns of elevational species richness often reflect the ecology of the taxonomic group [21, 22]. NTF at these sites are found in soil generally in low abundances and at low diversity in comparison to many other taxonomic groups. Considering the specialized feeding strategies and dependence on nematodes for food, NTF elevational diversity pattern may be driven by nematode abundance and diversity. Nematodes are the most abundant metazoans in the soil, highly diverse, and occupy numerous, often overlapping, ecological niches [23]. They are essential to soil processes (e.g., decomposition, nitrogen cycling), productivity, and may be used as bioindicators of soil condition [24]. Nematodes worldwide exhibit several elevation diversity patterns (e.g., irregular patterns, increases with elevation and mid-elevation peak [25–27]) and the nematode diversity or abundance at the two sites is largely unknown, so it is difficult to draw conclusions. However, abundance and richness are commonly predicted to be highest in the most productive environments [28]. On both mountain ranges, temperature decreases with increasing altitude [29], along with soil depth and soil nutrients, which should decrease productivity, and consequently, species richness with altitude. Rainfall and soil water availability generally exhibit a mid-elevation maximum in this region. As productivity in the soil is closely related to these basic limiting factors of life, we might predict productivity, and consequently nematode abundance and richness, to be highest at some intermediate elevation, but likely in the lower half, which could be driving the diversity and abundance of NFT.

Yet, it is impossible for us to discount the potential effects of other area-related factors, such as the species-area relationship (monotonic decreasing) mid-domain effects (unimodal mid-elevation peak), community overlap (unimodal mid-elevation peak) and ecotone effects [2]. The species-area relationship (SAR) asserts that as survey area decreases, such as the upper limits of the mountain, number of species encountered will correspondingly reduce [2]; the mid-domain effect (MDE) states that if all species ranges are scattered randomly between the limits of the top and bottom of a mountain, there will be a greater number of overlapping species in the mid-elevations as a consequence of the geometric limitations of the top of the mountain and valley bottom [30]; the community overlap hypothesis assumes that the transitional zone where upper mountain communities overlap with lower mountain communities a mid-elevation diversity maximum occurs. The ecotone effect predicts diversity peaks at ecotones, with higher peaks at more significant ecotones. Currently, there is mixed support for these theories, and little support for these predictions as a single predictor [2]. Because NTF abundance is so low in the environment, the importance of area driven factors is difficult justify. From the available evidence, we suggest the predominant factors underlying NTF elevational richness pattern appear to be climatic and ecological interactions specific to the taxon; however, much more ecological research is necessary to better isolate the principal drivers.

### Comparison of sampling modes

Some of the variation in elevational diversity patterns that has been reported in the literature is potentially an artifact of sampling patterns, scale of study and post-sampling treatment of data [4]; however, empirical examples demonstrating these problems are few. We found results of the elevational richness and frequency patterns of NTF depended on the sampling patterns (sampling modes), scale of study (total elevation range) and post-sampling treatment of data (‘removing samples with anthropogenic disturbance). These results support the argument of Lomolino and Mark [4]. In the distance sampling method, the OF of NTF conformed to mid-elevation peak pattern, and species richness met LPMP pattern; however, the elevation sampling methods obtained a totally different result that the OF and species number of NTF both showed a decreasing pattern. It suggests that sampling methods affect the observed pattern. Our results re-emphasize the importance of using robust sampling in developing species richness and OF models along environmental gradients.

Undoubtedly, in the process of studying the distribution patterns of species, increasing sampling efforts sees a consequent increase to a model’s accuracy [31]. However, when the sampling sites are unevenly distributed, the results will likely be biased. The equidistant sampling method along the sample line in Gaoligongshan resulted in sampling points that were not averagely distributed on the elevational gradient but concentrated in the middle altitude range, which probably caused the overestimation on the species richness of NTF in this area. The correlation analysis on the number of species and sample points indicated that they were strongly correlated with each other, thus this sampling pattern in which the sample points were concentrated in the middle altitude areas showed a mid-elevation peak pattern.

When using the evenly sampling method along the elevational gradient at Gaoligongshan, the elevational distribution of NTF showed a decreasing pattern, this method would not be affected by the distribution of sampling sites and the scale of studied region, thus the decreasing pattern was probably closer to the true situation of the elevational distribution of NTF. We used the same sampling method in Cangshan and got the same results that NTF OF showed a decreasing pattern. This demonstrated that our hypothesis was validated in different regions, and the elevation sampling method can resolve the observation biases and present more accurate results.

### Sampling range

When we truncated the sampling range to 2100–3500 m at Gaoligongshan where the samples were equidistantly collected along the sample lines (distance method), it was found that the original mid-elevation peak pattern (mid-peak pattern for OF, LPMP pattern for species number) changed to a decreasing pattern for the elevation sampling method. Previous studies on truncation and scale effects on the elevational distribution of species have been carried out. Truncating the low-altitude range of the studied region led to the changes of the elevational distribution of species from the mid-peak pattern to a decreasing pattern [18]. When the elevational gradient was entirely surveyed, the pattern was hump-shaped, changing eventually to a decreasing pattern as the scale of extent diminished. Likewise, the OF at the same altitude range of Cangshan (2100–3500 m) was also a decreasing pattern. This result further supports the view that truncating the elevation gradient (i.e., insufficient sampling range) significantly affects the overall elevational distribution pattern observed.

### Anthropogenic disturbance

Areas rich in biodiversity often overlap with areas of high human populations, and it’s generally accepted that human disturbance can affect the distribution of biodiversity [32–34]. Surprisingly the influence from human disturbance on species richness models has been long ignored. In recent years, some researchers have given this some attention, pointing out that human populations are generally based around low elevations, and therefore human disturbance is not evenly distributed across most elevations [30, 35].

The OF of NTF at Cangshan exhibited a decreasing pattern, while species number showed the LPMP pattern. The eastern slope of Cangshan below 2200 m is occupied by villages and farms, in west slope of Cangshan, below 2400 m is farmland. The Cangshan Nature Reserve not includes areas down to 2100–2300 m, so that areas outside the reserve are affected by human disturbance to varying degrees [36]. When omitting the data collected in the range of 2100–2300 m from Cangshan, we found both the OF and species number of NTF exhibited a decreasing pattern, which suggesting human disturbance affects the elevational richness pattern of NTF. The biodiversity in the low-altitude areas may have been artificially reduced due to human disturbances, thus creating the illusion of a mid-peak pattern. This may have been the reason why the species number of NTF at Cangshan showed a LPMP pattern when including the human-disturbed areas. Although we did not specifically quantify human disturbance in this study, our ability to alter the observed pattern by eliminating human disturbed sites highlights the importance of present and past human activities on patterns observed in nature.

### Prospects and suggestions

When we used different indicators (OF and species number) to identify microbial biodiversity on the altitude gradient in this study, the same set of studies presented different results, it probably meant that our previous studies did not reflect the full picture of biodiversity. Johnathan et al. [37] pointed out that because of multidimensional and scale-dependent characteristics of biodiversity, it would be better to describe its change from multiple perspectives. Multidimensionality made the study of biodiversity at different time and different space more challenging than other variables in ecology [38]. In some studies, elevational diversity patterns presented by different indicators (species number, species density, evenness, biodiversity index) are different [12, 39, 40], thus the distribution patterns shown by different biodiversity indicators seemed to be interlaced, and different indicators showed different dimensions and different levels of biodiversity, to describe the biodiversity more fully from multiple dimensions needs more research and exploration.

Despite the viewpoints from Nogues et al. [18] that the removal of high altitude areas had little effect on elevational richness pattern of species, the Three River Parallel Region in China is characterized by vast elevation span, climate and vegetation; the alpine mudstone beach and yearly snow-pack in the high-altitude areas may cause steep fall of biodiversity. In this study, the altitude range was not large enough to completely cover the whole range of “the Three Rivers Parallel Region” and to carry out the exploration of the impact on the elevational distribution in the high-altitude areas. Future research should also focus on the integrity of the altitude range and further extend the studied areas.

We also note that OF is frequently used in microbiological studies as an indicator of species density which was relatively less affected, but the large animals might be more affected if the number of species was used as an indicator, therefore, the species inconsistency which was in the studies on the elevational distribution may be related to sampling patterns. Of course, the environmental heterogeneity, caused by altitude, is more than the difference of temperature, precipitation and vertical area. In the future, we need to systematically carry out the research by integrating environment, biological groups, sampling patterns and data analysis to obtain the real situation of the elevational distribution of species, which is crucial to understand the forming mechanism, maintaining mechanism and large-scale distribution of microorganisms.

Based on our results, we believe the elevation interval method coupled with a rigorous sampling effort are best suited to richness studies of NTF and other taxon across environmental gradients. The results in this study suggested that future studies should address the sample-laying patterns and ensure not only the sampling evenness at each altitude, but also the consistency of the altitude range between the studied areas. A rarefaction curve should be employed to determine the rationality of the sampling, wherever possible, and at the same time, attention should also be paid to the impact of human disturbance along any environmental gradient measured.

## Conclusions

The NTF elevational richness pattern appears to be driven by climatic factors and taxon-specific ecological traits, however, the sampling pattern and range influenced the elevational richness patterns. The human interference in low-altitude areas and the multi-dimensionality of biodiversity itself also have some influence on the elevational distribution pattern, but the specific mechanism and degree of impact are not clear. The results suggested that future studies on the elevational gradients of species richness should address these factors and try to adopt the elevation interval method to reduce the observation bias.

## Methods

### Studied subjects

NTF are a type of predatory Eukaryotic microorganism that capture nematodes with specialized vegetative mycelia that function as trapping structures. These trapping structures include adhesive networks (*Arthrobotrys*), adhesive knobs and branches (*Dactylellina*), and constricting rings (*Drechslerella*). NTF are widespread, occupying a range of aquatic and terrestrial habitats, and are important natural biocontrol agents [41], making NTF ideal for ecological research.

### Studied areas

Two sites were selected in the Three Parallel Rivers region of Yunnan, China. Gaoligongshan (24°56′–28°23′N, 98°08′–98°53′E) is an extensive mountain chain located along the Sino-Burmese border in northwestern Yunnan province, separating the Nujiang and Dulongjiang River catchments. The site was in the Gaoligongshan National Nature Reserve, Dulong Nationality Autonomous County. Cangshan (25°33′–25°59′N, 99°54′–100°12′E) is a mountain range in the Yuengling Range of the Hengduan Mountains in the Langcang River catchment. Both sites are situated at the intersection of the south subtropical and mid-subtropical climate zones and exhibit distinct climate and biological zones along an altitudinal gradient.

### Sample collection

**Fig. 7:**
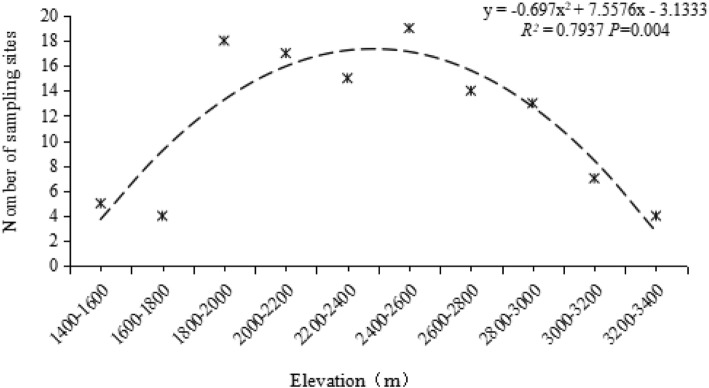
Species accumulation curve of Gaoligongshan (distance interval method, elevation interval method) and Cangshan. Both in Cangshan and the two sampling modes of Gaoligongshan, with the increase of the sampling point, the species accumulates continuously, and the final accumulation curve tends to be leveled, so the sampling efforts of each sampling mode in both regions are reasonable

Soil was collected using two modes (“distance interval method”, “elevation interval method”) between 1400 m and 3400 m at Gaoligongshan: (1) five bulked soil samples were collected from 116 sites at 1 kilometer intervals along the Dulong Jiang Highway (Fig. 7), 65 sites were located on the western face and 51 were located on the eastern face; (2) five bulked soil samples were collected from 40 sites at 100 m elevation intervals, 20 sites were located on the western face, and 20 were located on the eastern face. At Cangshan, five soil samples were collected from 30 sites at 100 m elevation intervals between 2100 and 3500 m. Fifteen sites were located on the western face, and 15 were located on the eastern face of Cangshan. Soil samples were stored in the laboratory at 4 °C.

### Preparation of culture medium

A corn meal agar medium (CMA, 20 g cornmeal, 20 g agar, 1000 ml water, boil cook cornmeal for 30 min, filter through cheesecloth, sterilized by autoclaving at 121 °C for 30 min) was used to isolate, purify and identify NTF. A potato dextrose agar medium (PDA, 200 g peeled potato, 20 g agar, 20 g glucose, 1000 ml water, boil potatoes for 30 min, filter through cheesecloth, sterilized by autoclaving at 121 °C for 30 min) was used as an enrichment culture to extract DNA. An oatmeal medium (10 g oat meal in 23 ml tap water, sterilized by autoclaving at 121 °C for 30 min) was used to cultivate and reproduce bait nematodes. A beef extract-peptone medium (3 g beef extract, 10 g peptone, 5 g NaCl, 20 g agar, 1000 ml water, pH 7.4–7.6) was used to sterile test the strains preservation tubes.

### Preparation of bait nematodes

The most common nematode used in studies of nematophagous fungi is a free-living soil nematode *Panagrellus redivivus* which is a well known commercially produced nematode used as a food source for fish larvae and can be bought online (http://www.atcc.org/) [42]. *P. redivivus* does not lay eggs, but the juveniles hatch internally. They have a short life cycle which has four larvalstages before becoming adults. The first larval stage is intrauterine, but the remaining stages are free-living [43]. This nematode is easy to rear in large quantities in culture and has a high fecundity.

Culture free-living nematodes with oatmeal medium for reproduce more nematodes. After 7–10 days of reproduction at room temperature, *P. redivivus* can be separated from the oatmeal by using a Baermann-funnel which allows active nematodes to pass through a filter [44, 45]. Nematodes were added to sterile water to put into suspension. Aliquots (0.01 ml) of the nematode suspension are placed on slides and gently heated to immobilize the nematodes which are then counted at 60 × magnification to estimate the population density [41].

### The isolation, purification and strains preservation of NTF

NTF were isolated from the soil using the soil sprinkling technique and purified using the single spore isolation method [41]. Soil samples (~ 2.5 g) were sprinkled on CMA medium with 5000 bait nematodes. This was repeated three times for each soil sample. After 1 week of at room temperature, microscopic examination of cultures was started using stereomicroscope. NTF were identified according to the morphology of conidia and conidiophores. Monitoring was continued for 4 weeks using the single spore isolation method. Different NTF were selected to introduce to CMA media and cultured in a 26.5 °C incubator for 7 days. The single conidia were separated 1–3 times until a pure culture was obtained.

Strain preservation tubes (soil storage method) were prepared by placing soil samples over mesh sieves (0.150 mm). Treated soil was placed in 1.8 ml frozen deposit tubes (1/3 volume), along with 500 μl distilled water to make the soil moist. Strain preservation tubes were sterilized by autoclaving at 121 °C for 30 min, placed overnight in a 37 °C constant (drying) temperature incubator and re-sterilized for 30 min (sterilized by autoclaving at 121 °C for 30 min). After the extraction of sterilized strains preservation tubes for sterile testing, one of ten tubes were pulled out and placed into the beef extract-peptone medium and placed in a 37 °C culture for 48 h. If there was any contamination by bacteria, then the tubes need to re-sterilized, and tested until shown to be sterile.

To store samples, a 7 mm internal diameter sterile perforator was used to perforate a pure culture of NTF. Sterile toothpicks were used to pick 3–5 cultures into the prepared preservation tubes, film-sealed, and stored at constant humidity (low) at 4–18 °C.

### Identification of NTF

Morphological identification was carried out according to the classification system and species description recorded in *NTF* [41]. Compared the conidia, conidiophores and nematode-trapping devices type with the species description in the NTF. The total DNA of fungi was extracted and the ITS (internal transcribed spacer region of the ribosomal RNA gene) and TUB (beta-tubulin gene) were sequenced. Species identification was carried out by sequence homologous analysis. Finally, the results of the comparison of the NTF form, the nematode-trapping devices type and the gene sequence identified the NTF to species. In this study, the cultures of the same species isolated from different samples are recorded as different strains.

Strains were resurrected by placing the cultures in the preservation tube in the center of CMA medium using a sterile toothpick and cultured in a 26.5 °C incubator for 7 days. Resurrected strains were used for subsequent identification. A 1.5 cm × 1.5 cm observation chamber was cut into the CMA medium center using a sterile scalpel [46]. A sterile cover glass was inserted at the edge of the observation chamber [46]. The NTF was inoculated at the edge of the observation chamber and cultured in a 26.5 °C incubator for 7 days. After the observation chamber and the cover glass were filled with hyphae, slides were made and placed under Olympus BX51 microscope (Olympus Corporation, Japan) for morphological analysis.

To observe the type of nematode-trapping devices, observation chambers with NTF hyphae was used to induce the production of nematode-trapping devices [47]. About 100 bait nematodes were added to the observation chamber, and monitored intermittently for 24–48 h for the formation of predatory organs.

To extract the total DNA, a PDA medium was used as enrichment culture for the NTF, and DNA was extracted using the CTAB method. Polymerase chain reaction (PCR) was used to amplify the ITS and TUB of the strains, using ITS1, ITS4, and Bt2a, Bt2b, respectively, as primers (10 min pre-denaturation at 95 °C, followed by 35 cycles of 1 min denaturation at 95 °C, 1 min annealing at 51 °C, 2 min extension at 72 °C, and a final extension of 10 min at 72 °C) [48, 49]. PCR products were sent to BioSune Biotechnology (ShangHai) Co., Ltd. to sequence the ITS and TUB genes forward and reverse with the same primer.

The Nucleotide BLAST on NCBI (National Center for Biotechnology Information Search database) was used to compare our sequences with GeneBank’s sequences and performs homologous analysis.

### Data treatment

Species accumulation curves of two sampling modes, Cangshan and Gaoligongshan, were plotted, where sample data was amalgamated into 200 m elevation intervals to calculate the detection rate and the number of species. Curve fitting and regression analyses were completed using SPSS software, using the most statistically significant and best-fitted model. Pearson correlation analysis was carried out on the number of samples and species under distance interval method, and the detection rate and the number of species in the two regions after the removal of human interference. Occurrence Frequency, OF (%), was calculated as: number soil samples per individual species/total soil samples × 100%.

The low-altitude areas (< 2300 m) of the Cangshan site have been disturbed through human development (i.e., intensive agriculture, villages, roads, etc.). To test natural diversity patterns NTF in the environment, we removed data from areas severely affected by human disturbance by using data only collected from within the protected nature reserve (> 2300 m), and then compared NTF elevational richness patterns at Cangshan and Gaoligongshan using rarefaction curves and correlation analysis.

## Data Availability

The dataset analyzed during the current study is available from the corresponding author on reasonable request.

## Abbreviations

CMA: Corn meal agar medium
ITS: Internal transcribed spacer region of the ribosomal RNA gene
LEP: Low-elevation plateau
LPMP: Low-elevation plateau with a mid-peak
NCBI: National Center for Biotechnology Information Search database
NTF: Nematode-Trapping Fungi
OF: Occurrence frequency
PDA: Potato dextrose agar medium
TUB: Beta-tubulin gene
